# lncRNA H19 facilitates the proliferation and differentiation of human dental pulp stem cells via EZH2-dependent LATS1 methylation

**DOI:** 10.1016/j.omtn.2021.04.017

**Published:** 2021-04-24

**Authors:** Zhen Du, Xiaoming Shi, Aizhong Guan

**Affiliations:** 1 Department of Stomatology, Linyi People’s Hospital, Linyi 276000, Shandong Province, P.R. China

**Keywords:** human dental pulp stem cells, long non-coding RNA, H19, LATS1, EZH2, differentiation

## Abstract

Human dental pulp stem cells (hDPSCs) have been recognized as a candidate cell source for tissue engineering. Long non-coding RNAs (lncRNAs) are differentially expressed in inflamed human dental pulp tissues. The present study is aimed at investigating the role of lncRNA H19 in the differentiation potential of hDPSCs. hDPSCs were successfully isolated and cultured, followed by conducting gain and loss-of-function experiments on lncRNA H19 and large tumor suppressor 1 (LATS1) to elucidate their respective biological functions in hDPSCs. lncRNA H19 was able to promote, whereas LATS1 was found to inhibit the differentiation, proliferation, and migration capabilities of hDPSCs. LATS1 was found to activate the Hippo-Yes-associated protein (YAP) signaling pathway by decreasing levels of YAP and Tafazzin (TAZ). The effects of lncRNA H19 on hDPSCs were achieved by repressing LATS1 through enhancer of zeste homolog 2-induced trimethylation of histone 3 at lysine 27. Finally, hDPSCs overexpressing lncRNA H19 and/or LATS1 were transplanted into nude mice. It was shown that lncRNA H19 inhibited LATS1 to promote the production of odontoblasts *in vivo*. Taken together, lncRNA H19 serves as a contributor to the differentiation potential of hDPSCs via the inhibition of LATS1, therefore highlighting novel therapeutic targets for dental pulp repair.

## Graphical abstract

**Figure:**
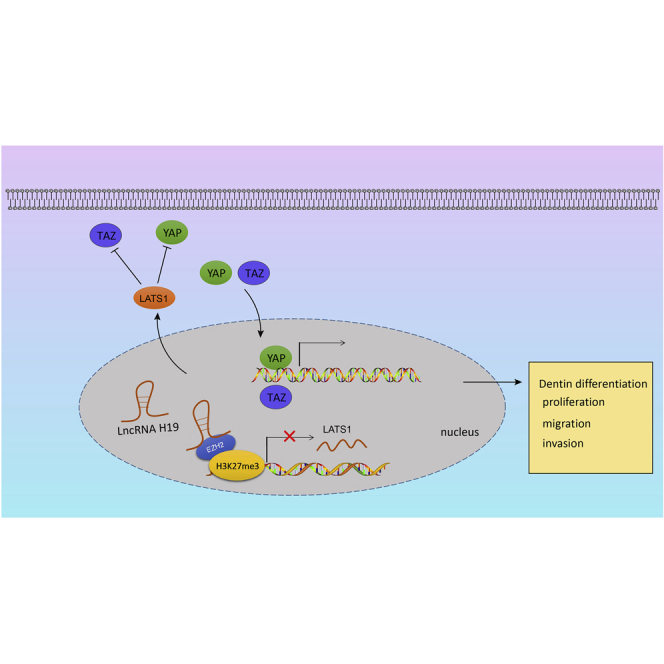

## Teaser

H19 promoted, while LATS1 inhibited, the differentiation, proliferation, and migration capabilities of hDPSCs. LATS1 activated the Hippo-YAP signaling pathway by decreasing YAP and TAZ. H19 serves as a contributor to the differentiation potential of hDPSCs via the inhibition of LATS1, therefore highlighting novel therapeutic targets for dental pulp repair.

## Introduction

Human dental pulp stem cells (hDPSCs) are primarily produced by pulp tissues of permanent and deciduous third molar teeth and primary incisors.^1^ hDPSCs are plastic-adherent cells with fibroblast-like morphology^2^ and possess a pronounced proliferation potential and differentiation capacity;^3^ they also possess clonogenic and proliferative abilities with differentiation potential into epithelial, neurogenic, adipogenic, osteogenic, and chondrogenic lineages.^4^ In addition, hDPSCs are a promising alternative source of mesenchymal stem cells (MSCs), as they express MSC makers and are easily isolated and obtained;^5^ therefore, hDPSCs may also be used for allograft transplantations.^6^ hDPSCs are increasingly becoming a potential source of autologous seed cells in relation to bone tissue engineering.^7^

Long non-coding RNAs (lncRNAs), non-coding RNAs (ncRNAs) of more than 200 nucleotides in length, have been implicated in regulating a wide array of cellular processes; for instance, cell differentiation and development, protein translation, RNA transcription, and DNA replication,^8^^,^^9^ particularly RNA polymerase II and the DNA duplex, regulate the transcription and expression of genes.^10^ Differentially expressed lncRNAs have been identified in inflamed human dental pulp tissues, suggesting the potential role of lncRNAs in the pathogenesis and progression of pulpitis.^11^ lncRNA H19, a paternally imprinted gene that encodes a 2.3-kb ncRNA, suppresses adipocyte differentiation in bone marrow MSCs (BMMSCs) via the epigenetic modulation of histone deacetylases.^12^ Furthermore, the silencing of lncRNA H19 has been reported to impair bone morphogenetic protein 9-induced osteogenic differentiation of MSCs *in vitro* and *in vivo*.^13^ lncRNA H19 has also been recently identified as an attractive therapeutic target for stem cell-based dentin regeneration by regulating the differentiation potential of hDPSCs.^14^ However, the detailed molecular mechanism underpinning this function still remains largely unknown.

The Hippo-Yes-associated protein (YAP) signaling pathway has been implicated in the development of various types of cancers and is capable of mediating the differentiation and self-renewal capabilities of multiple adult stem cells.^15^ Moreover, the Hippo-YAP signaling pathway plays a regulatory role in the proliferation of cells via contact inhibition and other properties of cellular physical states in tissues.^16^ YAP signaling has also been identified to affect adipo-osteogenic differentiation capabilities in human MSCs.^17^ The large tumor suppressor 1 (LATS1) is one of the central kinases of the Hippo-YAP signaling pathway, which can be phosphorylated and activated by Hippo, and functions as a cell-cycle modulator, tumor suppressor, and novel actin-binding protein.^18^ Activated LATS1 possesses the ability to degrade YAP by phosphorylating YAP; moreover, the demethylation of LATS1 can repress cell proliferation and promote cell apoptosis in renal cancer by decreasing the level of YAP.^19^ The absence of LATS1/2 triggers the activation of YAP/Tafazzin (TAZ), which has been implicated in the proliferation, migration, and differentiation capabilities of hDPSCs under the regulation of a static magnetic field.^20^ Based on the aforementioned literature, the current study aimed to elucidate the regulatory mechanism of lncRNA H19 in hDPSCs in an attempt to provide a theoretical foundation for solidifying the understanding of dental pulp repair.

## Results

### Successful isolation and culture of hDPSCs

**Figure 1:**
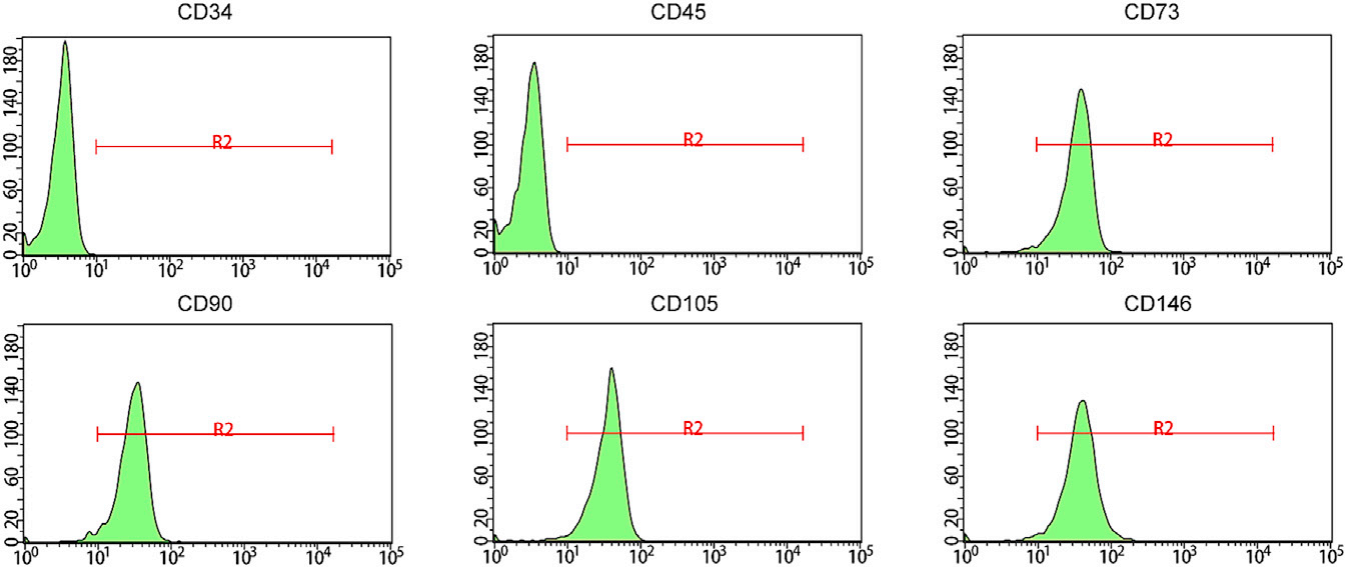
Expression of hDPSC surface markers (CD34, CD45, CD73, CD90, CD105, and CD146) detected by flow cytometry

The hDPSCs were initially isolated and cultured for the following experiments. Isolated hDPSCs were shuttle shaped and fiber shaped by having their morphologies altered (Figure S1A). Immunofluorescence was performed to detect the expression of hDPSC surface markers, the results of which suggested that CD44, CD90, CD105, and CD29 were positive in isolated hDPSCs (Figures S1B and S1C), whereas CD45 and CD11b were negative (Figure S1D). Additionally, flow cytometry demonstrated that cells were positive at CD73 (97.0%), CD90 (96.5%), CD105 (97.4%), and CD146 (97.0%) but negative at CD34 (0.6%) and CD45 (0.3%) (Figure 1), thus indicating the successful isolation and culture of hDPSCs.

### lncRNA H19 promotes odontoblast differentiation, proliferation, and migration of hDPSCs

**Figure 2:**
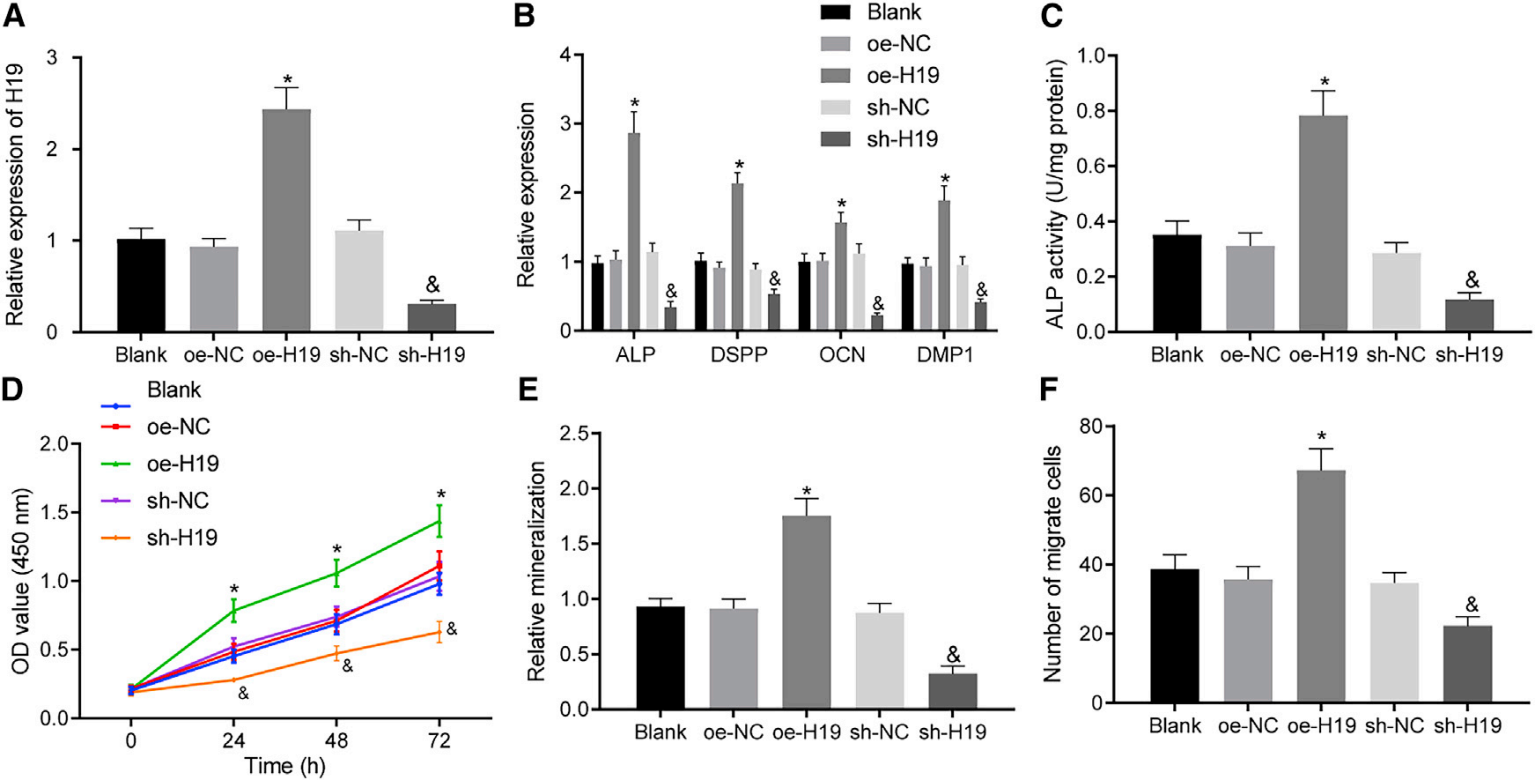
lncRNA H19 enhances odontoblast differentiation, proliferation, and migration of hDPSCs (A) Expression of lncRNA H19 determined by quantitative real-time PCR. (B) Expression of odontoblast differentiation-related genes (ALP, DSPP, OCN, and DMP1) determined by quantitative real-time PCR. (C) ALP staining detection. (D) Cell viability evaluated by CCK-8 assay. (E) The formation of mineralized nodules assessed by alizarin red staining. (F) Cell migration determined by Transwell assay. Each experiment was repeated 3 times independently. ∗p < 0.05, compared with the oe-NC group (hDPSCs transfected with oe-NC). &p < 0.05, compared with the sh-NC group (hDPSCs transfected with sh-NC).

Subsequently, we aimed to find out the effects associated with lncRNA H19 on odontoblast differentiation, proliferation, and migration of hDPSCs. The results of quantitative real-time polymerase chain reaction (PCR), western blot analysis, and alkaline phosphatase (ALP) activity detection illustrated that overexpressing lncRNA H19 had elevated the expression of odontoblast differentiation markers including ALP, dentin sialophosphoprotein (DSPP), osteocalcin (OCN), and dentin matrix protein 1 (DMP1) (Figures 2A, 2B, and S2A). Moreover, alizarin red staining was identified in hDPSCs overexpressing lncRNA H19, suggesting increased ALP activity and formation of mineralized nodules (Figures 2C, 2E, and S2B). Cell counting kit-8 (CCK-8) (Figure 2D) and Transwell assays (Figures 2F and S2C) revealed that the overexpression of lncRNA H19 had enhanced the viability and migration capabilities of hDPSCs. On the other hand, opposite changes were observed when H19 was silenced in hDPSCs. No significant differences were witnessed regarding the above-mentioned cellular interactions in hDPSCs without any treatment when compared with the transfection of negative control (NC) for the overexpressed (oe) plasmid (oe-NC) and short hairpin (sh)RNA targeting H19 (sh-NC), suggesting plasmid transfection had no impact on experimental data. Taken together, odontoblast differentiation, proliferation, and migration capabilities of hDPSCs were accelerated by lncRNA H19.

### LATS1 downregulates the expressions of YAP and TAZ in the Hippo-YAP signaling pathway

**Figure 3:**
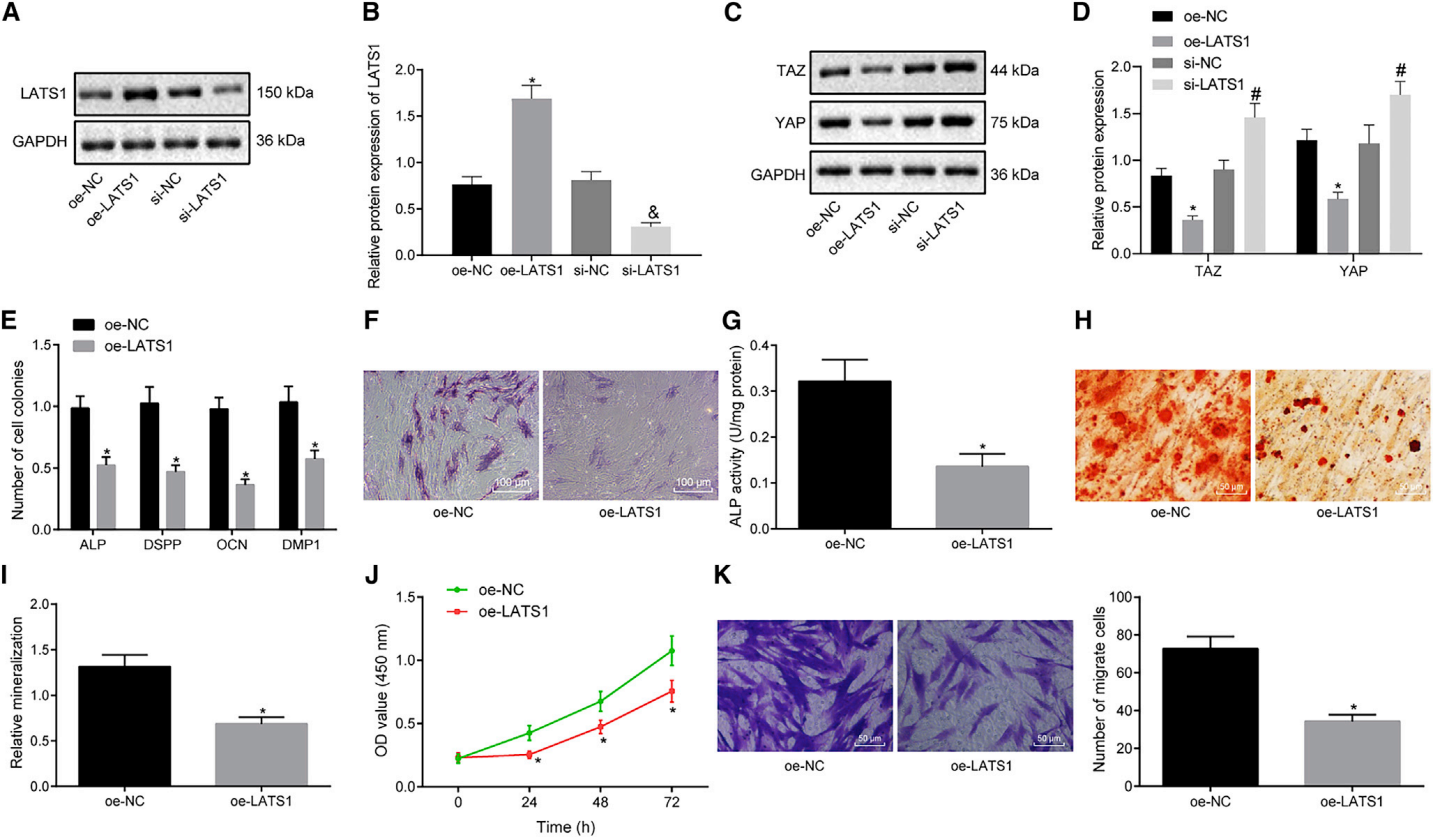
LATS1 inhibits the expression of YAP and TAZ of the Hippo-YAP signaling pathway (A) Protein band of LATS1 in hDPSCs measured by western blot analysis. (B) Protein level of LATS1 in hDPSCs measured by western blot analysis. (C) Protein bands of YAP and TAZ in hDPSCs measured by western blot analysis. (D) Protein levels of YAP and TAZ in hDPSCs measured by western blot analysis. (E) Expression of odontoblast differentiation-related genes (ALP, DSPP, OCN, and DMP1) in cells determined by quantitative real-time PCR. (F) ALP staining detection (×100). (G) ALP activity determination. (H) The formation of mineralized nodules examined by alizarin red staining (×200). (I) Relative mineralization of panel (H) graph. (J) Cell viability assessed by CCK-8 assay. (K) Cell migration evaluated by Transwell assay (×200). Each experiment was repeated 3 times independently. ∗p < 0.05, compared with the oe-NC group (hDPSCs transfected with oe-NC). #p < 0.05, compared with the si-NC group (hDPSCs transfected with si-NC).

In order to assess the impact of LATS1 on the differentiation, proliferation, and migration capabilities of hDPSCs, LATS1 was overexpressed/silenced by being transfected with oe-LATS1/small interfering (si)-LATS1 plasmids in hDPSCs (Figures 3A and 3B). Western blot analysis (Figures 3C and 3D) revealed that the overexpression of LATS1 had decreased the protein levels of TAZ and YAP, whereas the silencing of LATS1 led to opposing results. Quantitative real-time PCR (Figure 3E) indicated that the expressions of ALP, DSPP, OCN, and DMP1 were reduced after LATS1 was overexpressed. Lighter ALP staining (Figures 3F and 3G) and alizarin red staining (Figures 3H and 3I) results suggested that ALP activity was decreased with a reduced number of mineralized nodules in hDPSCs overexpressing LATS1. Repressed cell viability and migration capabilities were observed following the overexpression of LATS1 in hDPSCs, as illustrated by both CCK-8 (Figure 3J) and Transwell assays (Figure 3K). Altogether, LATS1 downregulated the expressions of YAP and TAZ of the Hippo-YAP signaling pathway.

### lncRNA H19 inhibits LATS1 expression via enhancer of zeste homolog (EZH2)-induced trimethylation of histone 3 at lysine 27 (H3K27me3)

**Figure 4:**
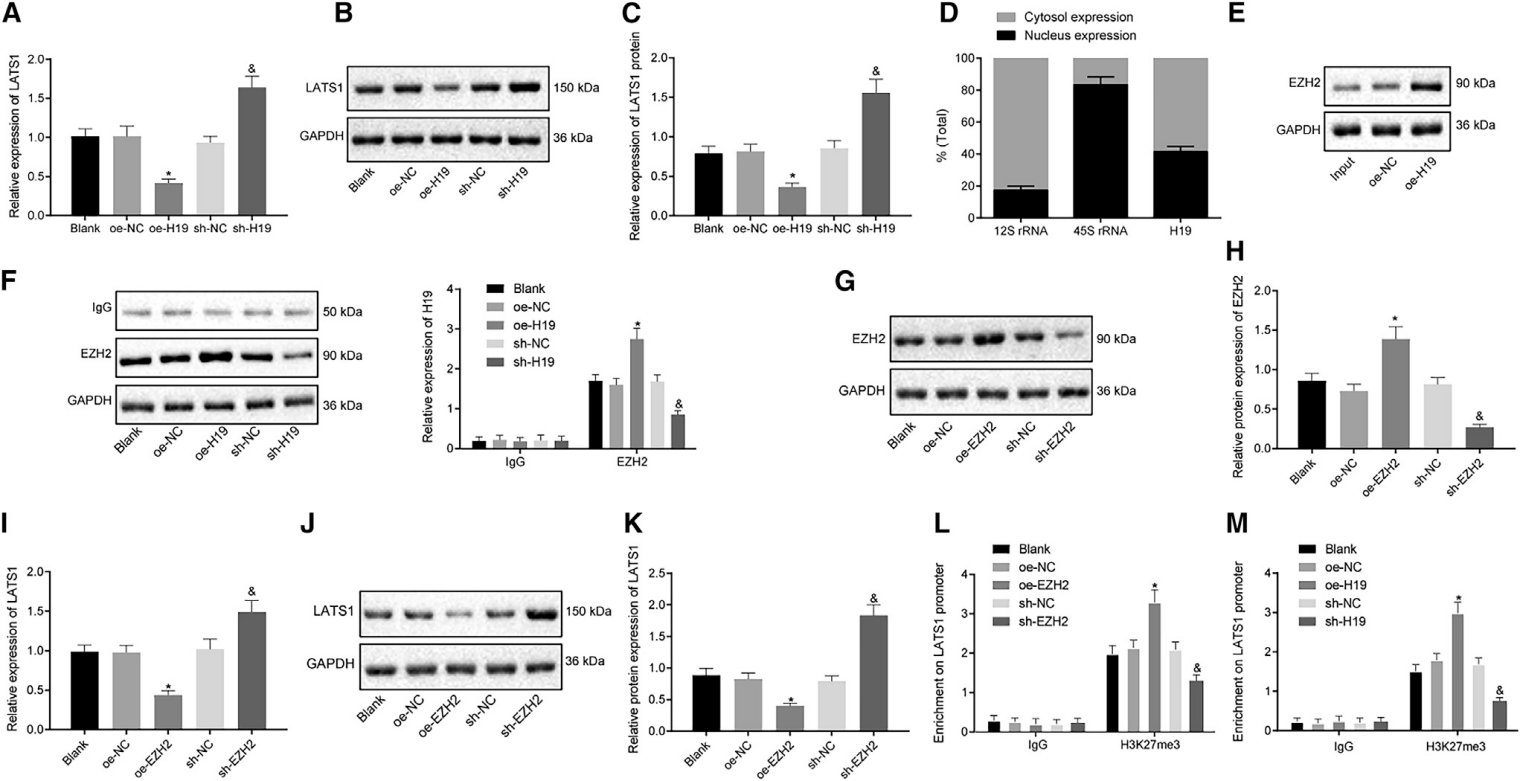
lncRNA H19 induces H3K27me3 and inhibits the expression of LATS1 by recruiting EZH2 (A) mRNA expression of LATS1 determined by quantitative real-time PCR after H19 was upregulated or silenced. (B) Western blots of LATS1 in hDPSCs. (C) Protein level of LATS1 in hDPSCs measured by western blot analysis. (D) Nuclear and cytoplasmic expression of lncRNA H19 in hDPSCs detected by quantitative real-time PCR. (E) Binding between lncRNA H19 and EZH2 assessed by RNA pull-down assay. (F) The enrichment of EZH2 by lncRNA H19 relative to IgG assessed by RIP assays. (G) Western blots of EZH2 in in hDPSCs. (H) Protein level of EZH2 in cells assessed by western blot analysis. (I) mRNA expression of LATS1 after upregulation or silencing of EZH2 determined by quantitative real-time PCR. (J) Western blots of LATS1 in hDPSCs after upregulation or silencing of EZH2 measured by western blot analysis. (K) Protein level of LATS1 in hDPSCs after upregulation or silencing of EZH2 measured by western blot analysis. (L) The enrichment of H3K27me3 on the LATS1 promoter after upregulation or silencing of EZH2 examined by ChIP assay. (M) The amount of H3K27me3 on the LATS1 promoter after upregulation or the silencing of lncRNA H19 detected by ChIP. Each experiment was repeated 3 times independently. ∗p < 0.05, compared with the oe-NC group (hDPSCs transfected with oe-NC). &p < 0.05, compared with the sh-NC group (hDPSCs transfected with sh-NC).

In order to explore the interactions between lncRNA H19 and LATS1, the expression of LATS1 in hDPSCs was determined by quantitative real-time PCR and western blot analysis after lncRNA H19 was either upregulated or silenced. The overexpression of lncRNA H19 led to a decrease in the expression of LATS1, whereas the silencing of lncRNA H19 led to an increase in the expression of LATS1 (Figures 4A−4C), highlighting that lncRNA H19 could negatively regulate the expression of LATS1. Next, in order to identify the mechanism of the regulation of LATS1 by lncRNA H19, we characterized the nuclear and cytoplasmic expressions of lncRNA H19, which displayed that lncRNA H19 expression was detected in both the nucleus and cytoplasm (Figure 4D). EZH2, commonly known as a methyltransferase, can be recruited by lncRNA H19 and induces H3K27me3 to regulate gene expression.^21^ Additionally, RNA pull-down and RNA binding protein immunoprecipitation (RIP) assays were performed to examine whether lncRNA H19 could recruit EZH2 in hDPSCs. Figure 4E depicts the binding between lncRNA H19 and EZH2 proteins; there was a significantly increased binding ability between lncRNA H19 and EZH2 proteins. The RIP kit (Millipore) was used for detecting binding between lncRNA H19 and EZH2 proteins as well; the results indicated that lncRNA H19 could recruit EZH2 protein. The overexpression of lncRNA H19 increased the enrichment of EZH2, whereas the silencing of lncRNA H19 impeded the enrichment (Figure 4F), indicating that lncRNA H19 could recruit EZH2 in hDPSCs. It was found that elevation of EZH2 had decreased the expression of LATS1 in hDPSCs; on the contrary, the inhibition of EZH2 had increased the expression of LATS1 (Figures 4G−4K). Additionally, the chromatin immunoprecipitation (ChIP) assay was carried out to assess the enrichment of H3K27me3 on the LATS1 promoter. As depicted in Figure 4L, the elevation of EZH2 potentiated the amount of H3K27me3 on the LATS1 promoter, whereas the silencing of EZH2 resulted in an inhibited amount of H3K27me3. These results proved that EZH2 had induced H3K27me3, thus inhibiting the expression of LATS1. Furthermore, after lncRNA H19 was overexpressed or silenced in hDPSCs, the concentration of H3K27me3 on the LATS1 promoter was determined again by ChIP. The results obtained demonstrated that the concentration of H3K27me3 on the LATS1 promoter was elevated by overexpressing lncRNA H19, whereas silencing lncRNA H19 reduced the concentration of H3K27me3 (Figure 4M). No significant difference was witnessed regarding the above-mentioned cellular behaviors in hDPSCs without any treatment when compared with that of the transfection of oe-NC and sh-NC, suggesting plasmid transfection had no impact on experimental data. The aforementioned findings suggested that lncRNA H19 recruited EZH2 to induce H3K27me3, thereby repressing the expression of LATS1.

### lncRNA H19 facilitates odontoblast differentiation, proliferation, and migration capabilities of hDPSCs via LATS1

**Figure 5:**
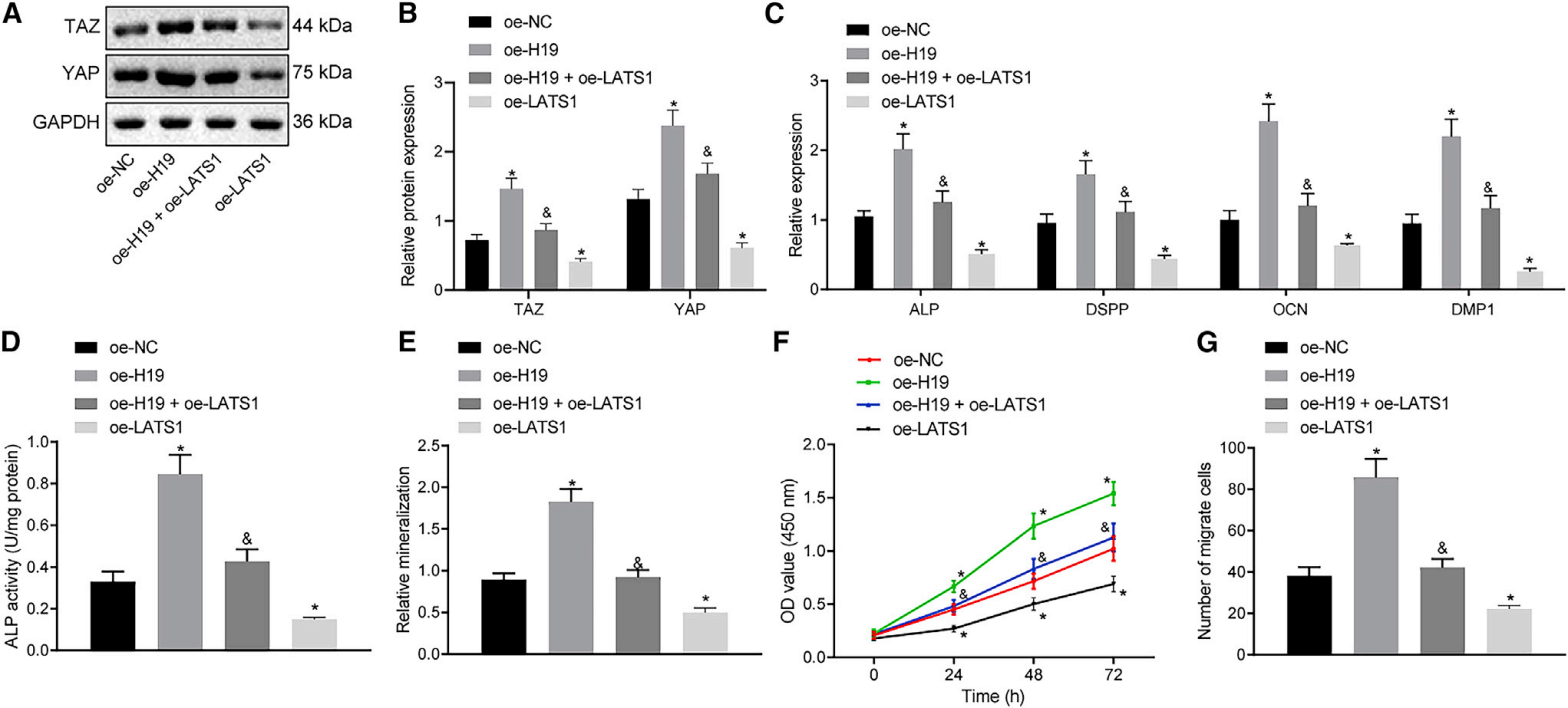
lncRNA H19 induces the differentiation, proliferation, and migration capabilities of hDPSCs through the regulation of LATS1 (A) Western blots of YAP and TAZ in hDPSCs determined by western blot analysis. (B) Protein levels of YAP and TAZ in hDPSCs determined by western blot analysis. (C) mRNA expression of ALP, DSPP, OCN, and DMP1 in hDPSCs detected by quantitative real-time PCR. (D) ALP activity determination. (E) The formation of mineralized nodules examined by alizarin red staining. (F) Viability of hDPSCs assessed by CCK-8 assay. (G) Migration of hDPSCs evaluated by Transwell assay. Each experiment was repeated 3 times independently. ∗p < 0.05, compared with the oe-NC group (hDPSCs transfected with oe-NC). &p < 0.05, compared with the oe-H19 group (hDPSCs transfected with oe-H19).

To further investigate the influence of lncRNA H19 and LATS1 on odontoblast differentiation, proliferation, and migration capabilities of hDPSCs, LATS1 and lncRNA H19 were overexpressed in hDPSCs. Quantitative real-time PCR and western blot analysis results initially indicated that the overexpression of lncRNA H19 had produced elevated protein levels of YAP and TAZ and mRNA levels of ALP, DSPP, OCN, and DMP1, all of which were decreased following the restoration of LATS1 (Figures 5A−5C). In addition to alizarin red staining, ALP staining and activity detection were also performed. The hDPSCs overexpressing lncRNA H19 displayed elevated ALP activity and increased formation of mineralized nodules, which following LATS1 overexpression, negated this argument (Figures 5D, 5E, S3A, and S3B). As depicted in Figures 5F, 5G, and S3C, cell viability, proliferation, and migration capabilities were elevated following the overexpression of lncRNA H19, which was repressed following the combined overexpression of lncRNA H19 and LATS1. Moreover, overexpressing LATS1 induced contrary results to those observed in the presence of overexpressed lncRNA H19 alone. Thus, based on the aforementioned results, we concluded that odontoblast differentiation, proliferation, and migration capabilities of hDPSCs, induced by lncRNA H19, could be inhibited by LATS1.

### lncRNA H19 promotes the production of odontoblasts

**Figure 6:**
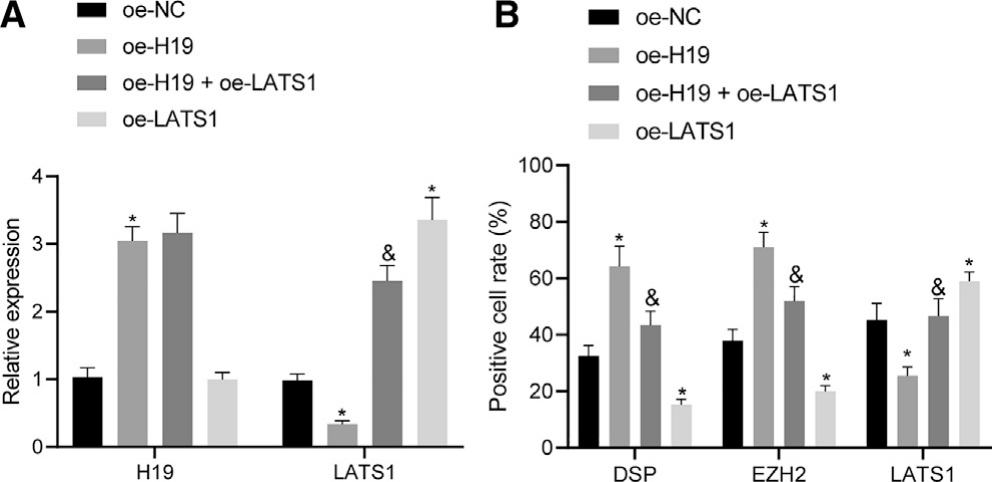
lncRNA H19 induces generation of odontoblasts Nude mice received the injection of hDPSCs transfected with oe-NC, oe-H19, oe-LATS1, or both oe-H19 and oe-LATS1. (A) The expression of lncRNA H19 and mRNA level of LATS1 determined by quantitative real-time PCR. (B) The expressions of DSPP, EZH2, and LATS1 in dentin and dental pulp-like tissues evaluated by immunohistochemistry. n = 12. ∗p < 0.05, compared with the oe-NC group (nude mice transplanted with oe-NC-transfected hDPSCs). &p < 0.05, compared with the oe-H19 group (nude mice transplanted with hDPSCs overexpressing H19).

In the final attempt to further elucidate the effect of lncRNA H19 on the differentiation of hDPSCs *in vivo*, hDPSCs overexpressing lncRNA H19 or lncRNA H19 with LATS1 were injected into nude mice. Quantitative real-time PCR demonstrated that the injection of hDPSCs overexpressing lncRNA H19 alone had increased the level of lncRNA H19 and decreased the level of LATS1, whereas the administration of hDPSCs overexpressing both lncRNA H19 and LATS1 restored the expression of LATS1, compared to the injection of hDPSCs overexpressing lncRNA H19 alone (Figure 6A). In addition, lncRNA H19 expression was not significantly different in response to overexpressed LATS1 alone, whereas LATS1 expression was increased. The control mice group exhibited cell polarization in connective tissues adjacent to the newborn matrix, resembling odontoblast-like cells, with a similar atypical dentin-pulp complex identified in a locally magnified region, as illustrated by hematoxylin and eosin (H&E) staining in Figure S4A. In nude mice injected with from nude mice injected with hDPSCs overexpressing lncRNA H19 alone, odontoblast-like characteristics were observed in cells arranged along dental pulps in paliform shape, with typical odontoblastic processes in the dentinal matrix. In the presence of overexpressed LATS1 alone, a dentin and dental pulp-like structure was generated in dental pulp-like tissues. Moreover, in the nude mice that received injections with hDPSCs overexpressing both lncRNA H19 and LATS1, dentin and dental pulp-like structures were visualized in dental pulp-like tissues, with odontoblast-like cells surrounded by dental pulp-like tissues. Immunohistochemistry was carried out to detect the expressions of DSP, LATS1, and EZH2 in dentin and dental pulp-like cells, revealing that the expressions of DSP and EZH2 were increased, and that of LATS1 was decreased following the administration of hDPSCs overexpressing lncRNA H19. On the other hand, the upregulation of LATS1 had reversed both the lncRNA H19-induced increase in expressions of DSP and EZH2 and the decline in expression of LATS1 (Figures 6B and S4B). The results suggested that the generation of odontoblasts was induced by lncRNA H19 and could be blocked by LATS1.

## Discussion

hDPSCs have been recently recognized as a kind of MSCs, owing to their strong proliferative and clonogenic potentials, which possess the ability to differentiate into different lineages.^22^ lncRNAs have been implicated in a vast array of biological processes that are significant to the development and differentiation processes, including the chondrogenic differentiation of human BMMSCs.^23^ Therefore, the current study was designed to investigate the regulatory effects associated with lncRNA H19 on hDPSC potential. Our results demonstrated that lncRNA H19 promoted the differentiation, proliferation, and migration capabilities of hDPSCs by repressing LATS1 through the Hippo-YAP signaling pathway.

A key initial finding of the current study was that lncRNA H19 accelerated the differentiation, proliferation, and migration capabilities of hDPSCs, in addition to increasing the expressions of ALP, DSPP, OCN, and DMP1. ALP has been identified as a valuable biomarker for clinical outcomes and diagnosis of disease.^24^ DSPP has been linked with dentin biomineralization, specifically, dentinogenesis imperfecta types II and III and dentin dysplasia type II, arising due to genetic deficiency in DSPP.^25^ OCN, a small non-collagenous protein, is primarily derived from osteoblasts and is overexpressed in most vertebrate bones.^26^ DMP1 represents a member of the dentin non-collagenous extracellular matrix proteins that trigger the cytodifferentiation of hDPSC into odontoblasts.^27^ lncRNA H19 has been reported to enhance the differentiation of osteogenic cells in human BMMSCs, via deriving microRNA-675 and through the regulation of the transforming growth factor-β1/Smad3/histone deacetylase signaling pathway.^28^ Moreover, lncRNA H19 can potentiate the osteogenic differentiation of BMMSCs, whereas its silencing reduces tension-induced osteogenic differentiation.^29^ The promotive effect of lncRNA H19 on the angiogenic capacity of BMMSCs has been recently demonstrated.^30^ A previous study has indicated that lncRNA H19 contributes to the accelerated odontogenic differentiation of hDPSCs through S-adenosylhomocysteine hydrolase-mediated, distal-less homeobox gene expression and methylation,^14^ a finding of which was consistent with the results of our study. Our *in vivo* experiments provided further validation regarding the accelerative role of lncRNA H19 in the production of odontoblasts.

Our study also demonstrated that lncRNA H19 was able to recruit EZH2 to promote H3K27me3, by which H19 inhibited the expression of LATS1 in hDPSCs. DNA methylation is closely linked to the changes in the nucleosome DNA scaffold that coordinates individual cellular gene expression and exerts its effect on cell differentiation.^31^ EZH2, the catalytic subunit of polycomb-repressive complex 2, has been widely recognized as a histone methyltransferase with a high degree of conservation that binds to histone H3K27; H3K27 methylation closely correlates with gene silencing.^32^ lncRNA H19 has been reported to accelerate bladder cancer metastasis by recruiting EZH2 and repressing the expression of E-cadherin.^33^ Furthermore, EZH2-dependent H3K27me3 has an impact on the decreased expression of inhibitor of DNA-binding/differentiation protein 4 in prostate cancer.^34^ EZH1 exerts pluripotency and sustains stem cell property by regulating histone H3K27 and reinforcing EZH2.^35^ A decline in LATS1 by FOXP4-AS1 has been reported to facilitate the progression of osteosarcoma by targeting lysine-specific demethylase 1 and EZH2.^36^ The downregulation of LATS1/2 by promoter methylation has been linked to the development of oral squamous cell carcinoma.^37^

We also identified that LATS1 had repressed the differentiation and migration capabilities of hDPSCs and lncRNA H19 by exerting its effect on hDPSCs by downregulating LATS1. Moreover, lncRNA H19 inhibited the Hippo-YAP signaling pathway via the repression of LATS1. The Hippo-YAP signaling pathway has been demonstrated to influence the size of tissues by directly modulating the maintenance and proliferation of stem cells;^38^ more specifically, Hippo and YAP have been shown to act as mediators for the differentiation of motor neurons of human pluripotent stem cells.^39^ Inhibited formation of the YAP/RUNX2 complex contributes to the enhancement of the osteogenic differentiation of MSCs.^40^ Moreover, the differentiation, migration, and proliferation capabilities of hDPSCs have been linked to YAP/TAZ activation mediated by a static magnetic field.^20^ As a common mediator of the Hippo-YAP signaling pathway, LATS1 negatively regulates the expression of oncogene YAP to activate this signaling pathway.^41^ Loss of YAP contributes to the suppression of adipo-osteogenic differentiation of human MSCs.^17^ A recent study concluded that lncRNA GHET1 promotes cell invasion and proliferation in non-small cell lung cancer by regulating the LATS1/YAP signaling pathway,^42^ the findings of which were consistent with our study. Uc.134, a novel lncRNA, elevates YAP^S127^ phosphorylation and represses LATS1 ubiquitination mediated by CUL4A to inhibit the progression of hepatocellular carcinoma.^43^ The overexpression of YAP1 and H19 is responsible for the pathogenesis of osteosarcoma,^44^ indicating a positive relationship between YAP1 and H19. Hence, the overexpression of H19 in hDPSCs resulted in elevated expressions of YAP and TAZ. However, the research is still at the preclinical stage, and more investigations on the involvement of the Hippo-YAP signaling pathway in hDPSCs are still warranted.

### Conclusions

**Figure 7:**
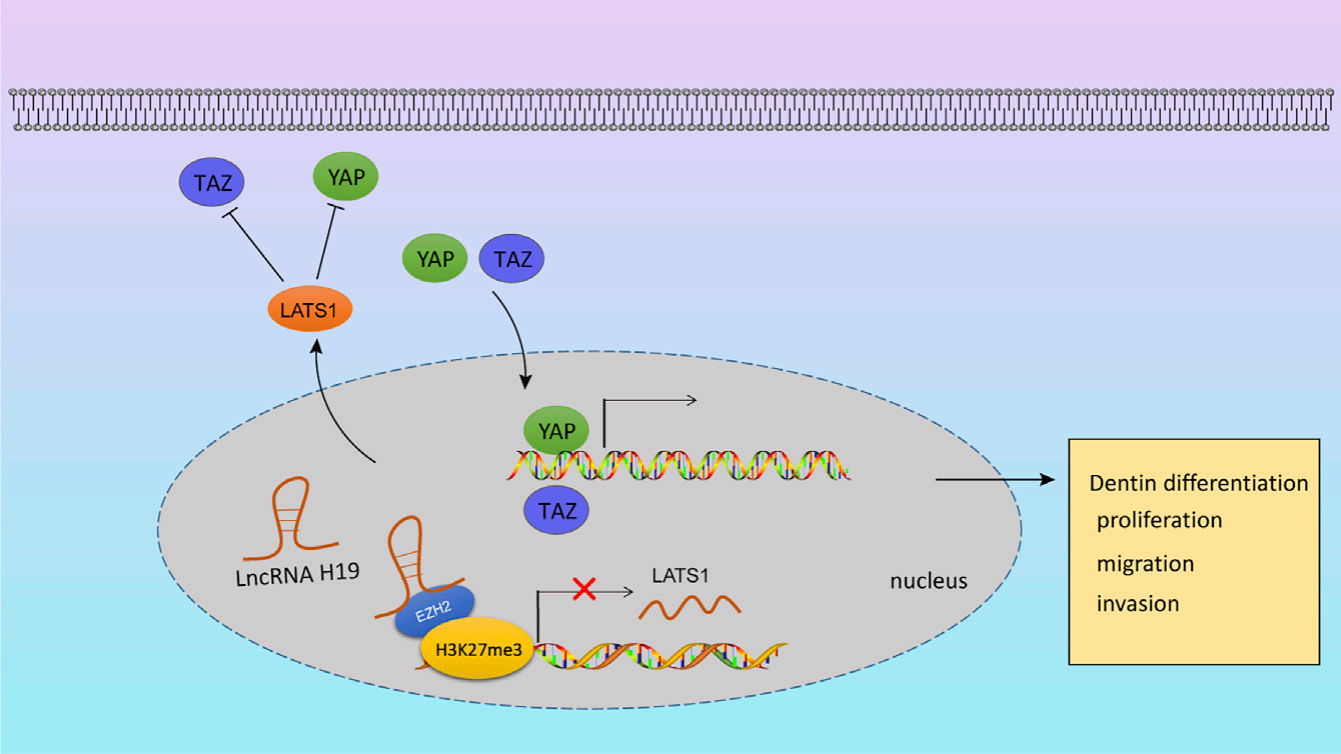
lncRNA H19 controls biological behaviors of hDPSCs through the LATS1-mediated Hippo-YAP signaling pathway lncRNA H19 recruits EZH2 to the LATS1 promoter region to induce H3K27me3, resulting in the inhibition of LATS1. The loss of LATS1 blocks the activation of the Hippo-YAP signaling pathway, thus enhancing dentin differentiation, proliferation, and migration capabilities.

Taken together, lncRNA H19 functioned as a promoter for dentin differentiation, proliferation, and migration capabilities of hDPSCs. This study has identified that lncRNA H19 recruits EZH2 to the LATS1 promoter region in order to enhance H3K27me3 and suppress the expression of LATS1, involving the Hippo-YAP signaling pathway (Figure 7). These findings provided evidence for linking the differentiation potential of hDPSCs to dental pulp repair and highlight potential novel therapeutic targets. Nonetheless, besides the marker expression of MSCs, the multipotential differentiation is still necessary to confirm MSC properties. Further studies on the Hippo-YAP signaling pathway balance mechanism in hDPSCs will be summarized in our next study.

## Materials and methods

### Ethics statement

The experimental procedures were approved by the Ethics Committee of Linyi People’s Hospital. Informed, written consents were obtained from each participant prior to sample collection; patients were informed before the collection of pulp tissue samples and the separation and culture of pulp stem cells. All animal experiments were performed in strict accordance with the recommendations in the *Guide for the Care and Use of Laboratory Animals* of the National Institutes of Health. The protocol of animal experiments was approved by the Institutional Animal Care and Use Committee of Linyi People’s Hospital. Extensive efforts were made to minimize the number of animals used and the pain inflicted on the animals.

### Isolation and culture of hDPSCs

The isolation and culture of hDPSCs were performed based on a previously reported method.^45^ Dental pulps were first isolated from healthy premolar teeth under aseptic conditions. Next, 2 mm of dental pulp tissues was separated from the crown and the root and washed twice with phosphate buffered saline (PBS) and trypsinized by collagenase I (BioSharp, Hefei, China) (3 mg/mL) and dispase (4 mg/mL) at 37°C for 70 min, followed with the addition of culture medium containing 15% fetal bovine serum (FBS), 100 U/mL streptomycin, and 100 U/mL penicillin and 2 mM of L-glutamine α-minimum essential medium. The cells were subsequently filtered through a 70-μm strainer and transferred into a centrifuge tube to centrifuge for 5 min. After the supernatant had been removed, the cells were suspended in a cell culture medium, inoculated into the culture dish at a density of 1 × 10^5^ cells/mL, and incubated at 37°C with 5% CO_2_; the culture medium was renewed every 3 days.

### Immunofluorescence

hDPSCs were fixed with 4% paraformaldehyde for 15 min and blocked with normal goat serum at room temperature for 30 min. The cells were then incubated with primary antibodies against CD44 (ab157107, 1:500; Abcam, Cambridge, UK), CD90 (ab133350, 1:100; Abcam), CD105 (ab221675, 1:1,000; Abcam, Cambridge, UK), CD29 (ab24693, 1:1,000; Abcam, Cambridge, UK), CD45 (ab10558, 1:1,000; Abcam, Cambridge, UK), and CD11b (ab8878, 1:200; Abcam, Cambridge, UK) overnight at 4°C. After cells were washed with PBS solution, they were re-probed with Alexa Fluor 488-labeled goat anti-rabbit immunoglobulin G (IgG) (ab150077, 1:200; Abcam) under complete darkness at 37°C for 1 h. The cells were subsequently left to stain with 5 μg/mL 4′,6-diamidino-2-phenylindole for 5 min and mounted at 4°C in complete darkness. The cells were then observed under a laser-scanning confocal microscope (ZEISS LSM 510 M ETA; Carl Zeiss, Jena, Germany) and imaged using NIS-Elements Viewer software at an emission wavelength of 519 nm. The nuclei were stained in blue fluorescence, whereas cells positive for CD44 and CD90 were stained in green fluorescence.

### Flow cytometry

Flow cytometry analysis was used to detect the surface markers of hDPSCs. A total of 1 × 10^6^ cells were trypsinized and incubated with anti-human CD90, CD73, CD105, CD146, CD34, and CD45 antibodies. All antibodies were purchased from BD Biosciences (USA). The flow cytometry analysis was performed on the FACSAria II flow cytometer (BD Biosciences, USA).

### Cell treatment

The Lipofectamine 2000 (Invitrogen, Carlsbad, CA, USA) reagent kit (11668019), purchased from Thermo Fisher Scientific (Waltham, MA, USA), was employed for cell transfection. Cells were transfected with lncRNA H19 overexpression plasmid (oe-H19), shRNA targeting lncRNA H19 (sh-H19), oe-LATS1, oe-EZH2, and sh-EZH2 and their NCs (oe-NC and sh-NC) based on the instructions provided. Part of hDPSCs remained untreated, thus serving as the blank control. After cells were transfected for 48 h, they were collected for the following experiments after the determination of transfection efficacy with quantitative real-time PCR and western blot analysis.

### Quantitative real-time PCR

TRIzol (Invitrogen) reagent was used to extract the total RNA content from hDPSCs, and NanoDrop 2000 micro-ultraviolet spectrophotometer (1011U; NanoDrop Technologies, Wilmington, DE, USA) was employed to determine the concentration and purity of the total RNA obtained. The extracted RNA was then reversely transcribed into complementary DNA (cDNA) in accordance with the instructions of the PrimeScript RT Reagent Kit (RR047A; Takara, Shiga, Japan). Quantitative real-time PCR was then performed using an ABI7500 qPCR instrument (Applied Biosystems, Foster City, CA, USA). With the use of glyceraldehyde-3-phosphate dehydrogenase (GAPDH) as an internal reference, the 2^−△△CT^ method was applied to calculate the relative expression of target genes.^46^ The primers for lncRNA H19, ALP, DMP1, OCN, DSPP, and LATS1 were all synthesized with the Takara PCR Amplification Kit (Table S1).

### Western blot analysis

The total protein content was extracted from hDPSCs using radio-immunoprecipitation assay (RIPA) cell lysis buffer, supplemented with phenylmethanesulfonylfluoride on ice for 30 min. The supernatant was collected following centrifugation at 4°C and 8,000 × *g* for 10 min. The total protein concentration was subsequently determined using a bicinchoninic acid reagent kit. A total of 50 μg protein was separated by sodium dodecyl sulfate-polyacrylamide gel electrophoresis and transferred onto polyvinylidene fluoride membranes, which were left to block with 5% skim milk at room temperature for 1 h. Afterward, the membranes were probed overnight at 4°C with diluted primary rabbit antibodies against LATS1 (ab70561, 1:5,000), YAP (ab205270, 1:1,000), EZH2 (ab186006, 1:500), TAZ (ab242313, 1:1,000), ALP (ab229126, 1:800), DSPP (ab272929, 1:1,000), OCN (ab93876, 1:500), DMP1 (ab103203, 1:1,000), and internal reference GAPDH (ab9485, 1:2,500). The membranes were later incubated with horseradish peroxidase (HRP)-labeled rabbit antibodies to IgG (ab97051, 1:2,000) for 1 h. The aforementioned antibodies were purchased from Abcam. The proteins were developed using an enhanced chemiluminescence reagent (BB-3501; Amersham, Arlington Heights, IL, USA), and band intensities were quantified using the Bio-Rad image analysis system (Bio-Rad, Hercules, CA, USA) and Quantity One version (v.)4.6.2 software. The relative expression was expressed as the ratio of the gray value of the target band to that of GAPDH.

### ALP activity detection

The hDPSCs were first treated with an osteogenic induction medium (OM) for 14 days. Afterward, the hDPSCs were left to stain using an ALP staining kit (1102-100; SiDanSai Biotechnology, Shanghai, China) for 15 min in complete darkness. The reaction was terminated by rinsing with distilled water 2 to 3 times. An inverted microscope (Carl Zeiss) was employed to observe and analyze staining, and an ALP activity detection kit (Nanjing Jiancheng Bioengineering Institute, Jiangsu, China) was used to determine the activity of ALP.

### Alizarin red staining

hDPSCs were treated with OM for 28 days. Next, hDPSCs were rinsed three times with 0.01 mol/L PBS solution and were left to fix with 95% ethanol for 15 min. The cells were left to stain with alizarin red for 5 min following fixation, rinsed with distilled water, dried, and mounted. Finally, the formed mineralized nodules were observed under an inverted microscope (Carl Zeiss).

### CCK-8 assay

The viability of the hDPSCs was assessed using a CCK-8 kit (CK04; Dojindo Laboratories, Kumamoto, Japan). The hDPSCs at the logarithmic growth phase were cultured in a 96-well plate at a density of 1 × 10^4^ cells/well for 24 h. After hDPSCs were transfected for 48 h, 10 μL of CCK-8 reagent was added at the following time points: 0 h, 24 h, 48 h, and 72 h, respectively, and the cells were cultured for an additional 3 h. The absorbance value of each well was then determined at a wavelength of 450 nm using a microplate reader. Finally, the results obtained were used to plot a cell growth curve.

### Transwell assay

hDPSCs at the logarithmic growth phase were inoculated into a 6-well plate. After transfection for 48 h, the cells were detached with 0.25% trypsin, centrifuged, and re-suspended in serum-free Dulbecco’s modified Eagle’s medium (DMEM) with the cell density adjusted into 3 × 10^5^ cells/mL. Next, 100 μL of cell suspension was added into the apical chambers of the 24-well Transwell chamber, after which, 500 μL of DMEM supplemented with 10% FBS solution was added to the basolateral chambers followed by incubation at 37°C with 5% CO_2_ for 24 h. After that, the chambers were washed twice with PBS, and the cells were fixed with formaldehyde for 10 min and stained with crystal violet dye for a further 10 min. Subsequently, the cells in the upper layer of chambers were wiped off carefully with a cotton swab, and the membrane was removed and sealed in a glass slide with a neutral resin, followed by cell counting under a microscope with 6 randomly selected fields.

### Fractionation of nuclear/cytoplasmic RNA

The nuclear and cytoplasmic RNA fractions were isolated according to the instructions of the PARIS Kit (Life Technologies, Gaithersburg, MD, USA). The hDPSCs were re-suspended in 500 μL of cell fractionation buffer and incubated on ice for 5 to 10 min. The cell lysate was centrifuged at 500 × *g* and at 4°C for 5 min. The resulting supernatant (cytoplasmic fraction) was harvested into a 2-mL sterile enzyme-free tube, followed by centrifugation. The pellet (nuclear fraction) was lysed using 500 μL of cell disruption buffer, followed by centrifugation. The cytoplasmic and nuclear fractions were separately rinsed in 500 μL 2 × lysis/binding solution, followed by centrifugation. The fractions were mixed with 500 μL absolute ethanol and then transferred into a filter cartridge. RNA from various fractions was harvested after being washed in Wash Solution I and 2/3. The expression of lncRNA H19 was determined by quantitative real-time PCR, with 45S rRNA as the internal control for nuclear RNA expression and 12S rRNA for the expression of cytoplasmic RNA. The primers used are illustrated in Table 1.

**Table 1:** Primer sequences for nuclear/cytoplasmic RNA quantification

Gene	Primer sequences
45S rRNA	F: 5′-GTGCCCTCACGTGTTTCACTTT-3′
	R: 5′-TAGGAGACAAACCTGGAACGCT-3′
12S rRNA	F: 5′-TCGATAAACCCCGCTCTACCT-3′
	R: 5′-TGGCTACACCTTGACCTAACGTT-3′

F, forward; R, reverse.

### RNA pull-down assay

lncRNA H19 RNA fragments were obtained *in vitro* after being treated with T7 RNA polymerase (Ambion, Austin, TX, USA), which was then treated with DNase I and purified using the RNeasy Mini Kit (QIAGEN, Hilden, Germany). The 3′ untranslated region of the purified RNA was biotin labeled with RNA labeling compounds (Ambion). Next, 1 μg labeled RNA was heated in a RNA structure buffer (10 mM Tris, pH 7, 0.1 mol/L KCl, 10 mM MgCl_2_) at 95°C for 2 min, incubated on ice for 3 min, and allowed to stand at room temperature for 30 min. Afterward, the hDPSCs were lysed in cell lysis buffer (Sigma-Aldrich, St. Louis, MO, USA) at 4°C for 1 h, followed by centrifugation. The supernatant was then transferred into an RNase-free centrifuge tube. Biotinylated RNA (400 ng) was subsequently mixed with 500 μL of RIP buffer and then incubated with cell lysate at room temperature for 1 h, which was later incubated with streptavidin magnetic bead-protein complexes at room temperature for 1 h. Finally, the beads were washed five times with RIP buffer and boiled with 5 × loading buffer at 95°C for 5 min. Western blot analysis was performed in order to determine the level of eluted EZH2 protein.

### RIP assay

An RIP kit (Merck Millipore, Billerica, MA, USA) was utilized to detect the binding of lncRNA H19 and EZH2 protein. The cells were then lysed with RIPA lysis buffer (P0013B; Beyotime Institute of Biotechnology, Shanghai, China) for 5 min on ice. The supernatant was collected after centrifugation at 4°C for 10 min. Next, 50 μL magnetic beads were re-suspended in 100 μL RIP Wash Buffer and incubated with 5 μg of antibodies. Afterward, magnetic bead-antibody compounds were re-suspended in 900 μL RIP Wash Buffer and incubated with 100 μL cell lysate overnight at 4°C. After the eluted magnetic bead-protein complexes were treated with protease K, RNA was extracted and quantified by quantitative real-time PCR. The antibodies used in RIP assay included rabbit antibodies against EZH2 (ab186006, 1:100; Abcam) and IgG (ab109489, 1:100; Abcam) serving as NC.^47^ The used primers are displayed in Table 2.

**Table 2:** Primer sequences for RIP assay

	Primer sequences
H19	F: 5′-GCACCTTGGACATCTGGAGT-3′
	R: 5′-TTCTTTCCAGCCCTAGCTCA-3′
GAPDH	F: 5′-ATGGAGAAGGCTGGGGCTC-3′
	R: 5′-AAGTTGTCATGGATGACCTTG-3′

### ChIP assay

When the cell confluence reached 70%−80%, the hDPSCs were left to fix with 1% formaldehyde at room temperature for 10 min. Next, the crosslinked chromatin was sonicated 15 times at an interval of 10 s. After centrifugation at 30,237 × *g* and at 4°C, the supernatant was collected and incubated with mouse antibodies against IgG (ab205719, 1:100; Abcam) in the NC group and mouse antibody against H3K27me3 (ab6002, 1:100; Abcam) overnight at 4°C, respectively. Protein agarose/sepharose was utilized to precipitate endogenous DNA-protein compounds. DNA was then allowed to de-crosslink overnight at 65°C and was extracted with phenol/chloroform. Finally, quantitative real-time PCR was performed to determine the level of target DNA.^48^

### Xenograft tumor in nude mice

A total of 48 BALB/c nude mice (aged 4 weeks; weighing 18−22 g; both sexes) were purchased from Hunan SJA Laboratory Animal (Hunan, China) (http://www.hnsja.com/) and raised under specific pathogen-free conditions. hDPSCs (1 × 10^7^ cells/mL) were confirmed to stably express lncRNA H19 or LATS1; afterward, ceramic bovine bone composite scaffolds were subcutaneously transplanted into the back region of the nude mice. After 8 weeks had elapsed, the dental pulp-like tissues, which were later embedded with paraffin and sectioned, were collected and decalcified with 10% ethylene diamine tetra acetic acid for 1−2 days. Next, the tissues were stained with H&E, observed, and photographed under a microscope. Immunohistochemistry was subsequently conducted to determine DSPP levels after incubation with primary rabbit antibodies against DSPP (ab216892, 1:500; Abcam), EZH2 (ab227648, 1:500; Abcam), and LATS1 (ab234820, 1:500; Abcam) and secondary antibody HRP-labeled goat anti-rabbit IgG (ab6721, 1:500; Abcam) at room temperature for 30 min, followed by microscopic observation and image capturing. ImageJ software was used to calculate the number of positive cells in each picture, calculate the proportion of positive cells, and finally analyze the difference between the proportion of positive cells in each group.

### Statistical analysis

Data analyses were performed using SPSS 19.0 (/20.0/21.0/22.0) software (IBM, Armonk, NY, USA). Measurement data were expressed as the mean ± standard deviation. If the data were consistent with both normal distribution and homogeneity of variance, then an unpaired t test was applied for data comparison between two groups. One-way analysis of variance (ANOVA) was conducted for data comparisons among multiple groups, followed by Tukey’s post hoc test for multiple comparisons. Repeated measures of ANOVA were utilized for the comparison of data at different time points, followed by a Bonferroni post hoc test for multiple comparisons. p < 0.05 was considered to be indicative of statistical significance.

## Supplemental information

Document S1. Figures S1–S4 and Table S1

Document S2. Article plus supplemental information
