# Supplementary material for: lncRNA H19 facilitates the proliferation and differentiation of human dental pulp stem cells via EZH2-dependent LATS1 methylation
Source: Mol Ther Nucleic Acids. 2021 Apr 24;25:116–26. doi: 10.1016/j.omtn.2021.04.017 (PMC8339349; doi:10.1016/j.omtn.2021.04.017)
Supplement: Document S1. Figures S1–S4 and Table S1 [file mmc1.pdf]

OMTN, Volume *25*

Supplemental information

lncRNA H19 facilitates the proliferation and differentiation of human dental pulp stem cells via EZH2-dependent LATS1 methylation

Zhen Du, Xiaoming Shi, and Aizhong Guan

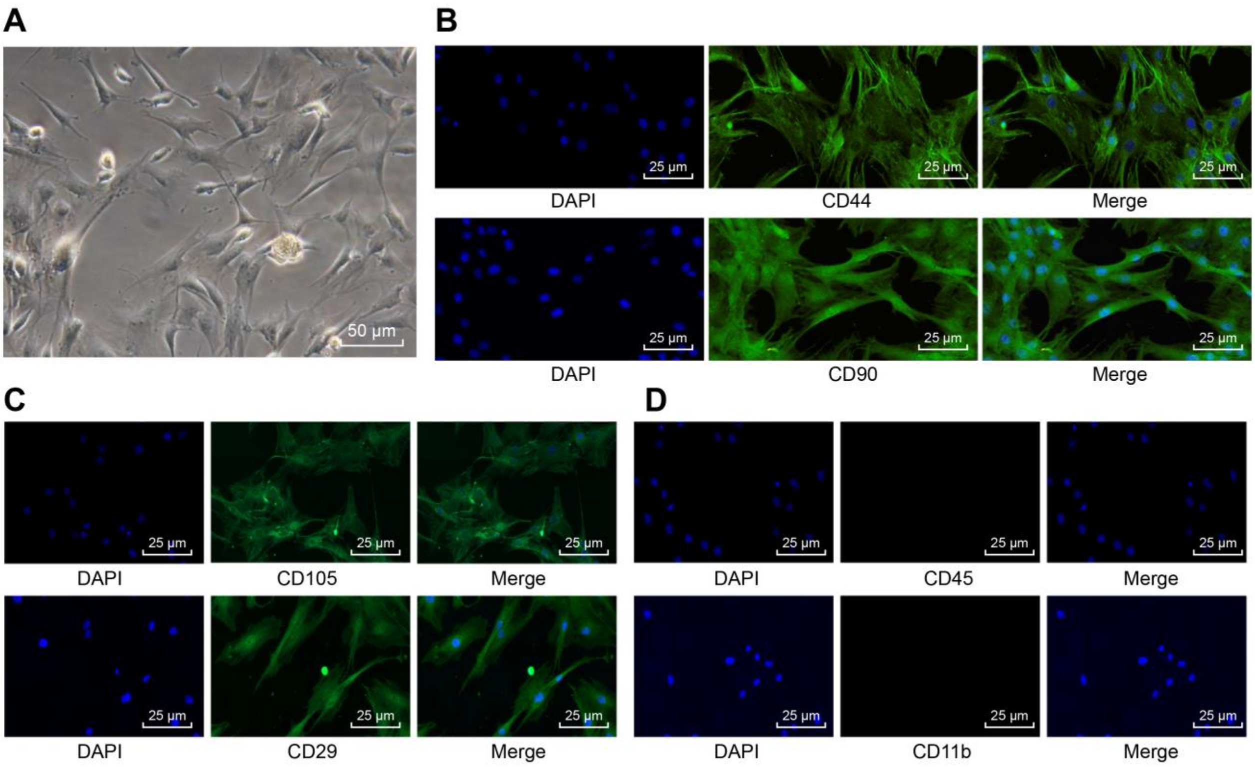

Figure S1 Successful isolation and culture of hDPSCs. A, morphology of hDPSCs observed under an inverted microscope (× 200). B, expression of hDPSC surface markers (CD44 and CD90) detected by immunofluorescence (× 400). C, expression of hDPSC surface markers (CD105 and CD29) detected by immunofluorescence (× 400). D, expression of hDPSC surface markers (CD45 and CD11b) detected by immunofluorescence (× 400).

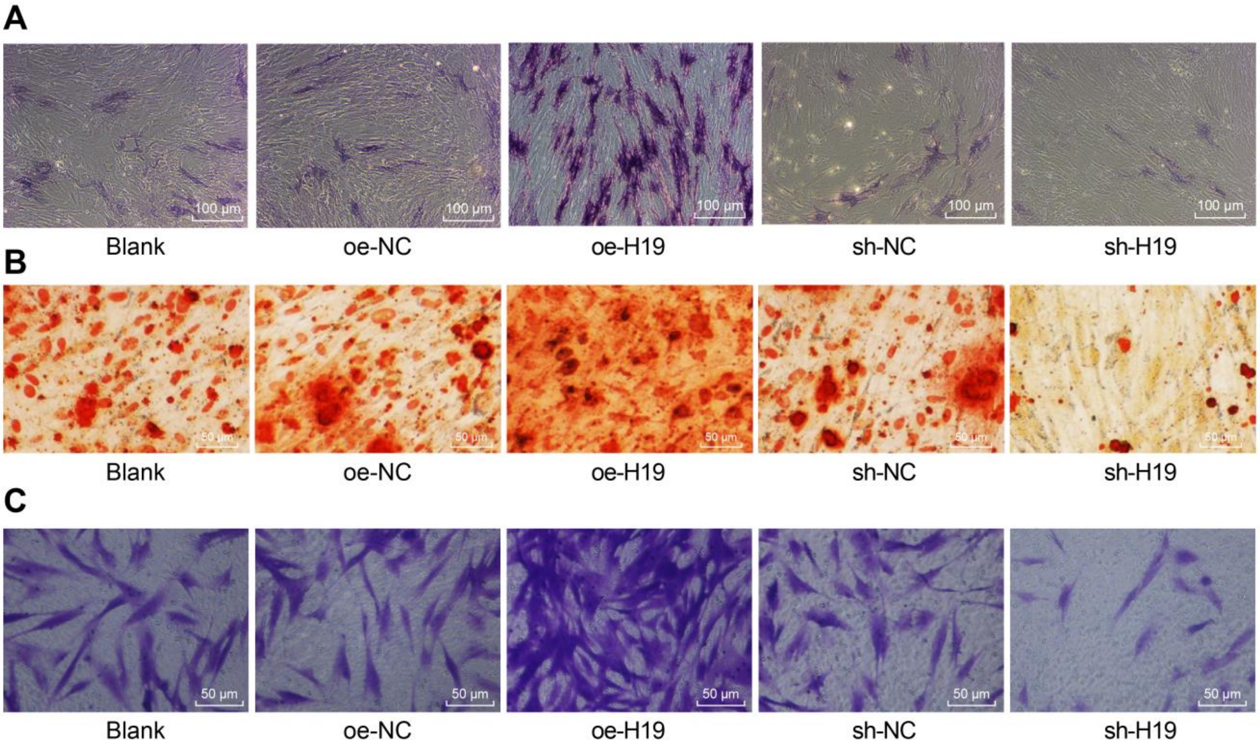

Figure S2 A, ALP staining detection (×100). B, the formation of mineralized nodules assessed by Alizarin red staining (×200). C, cell migration determined by Transwell assay (×200).

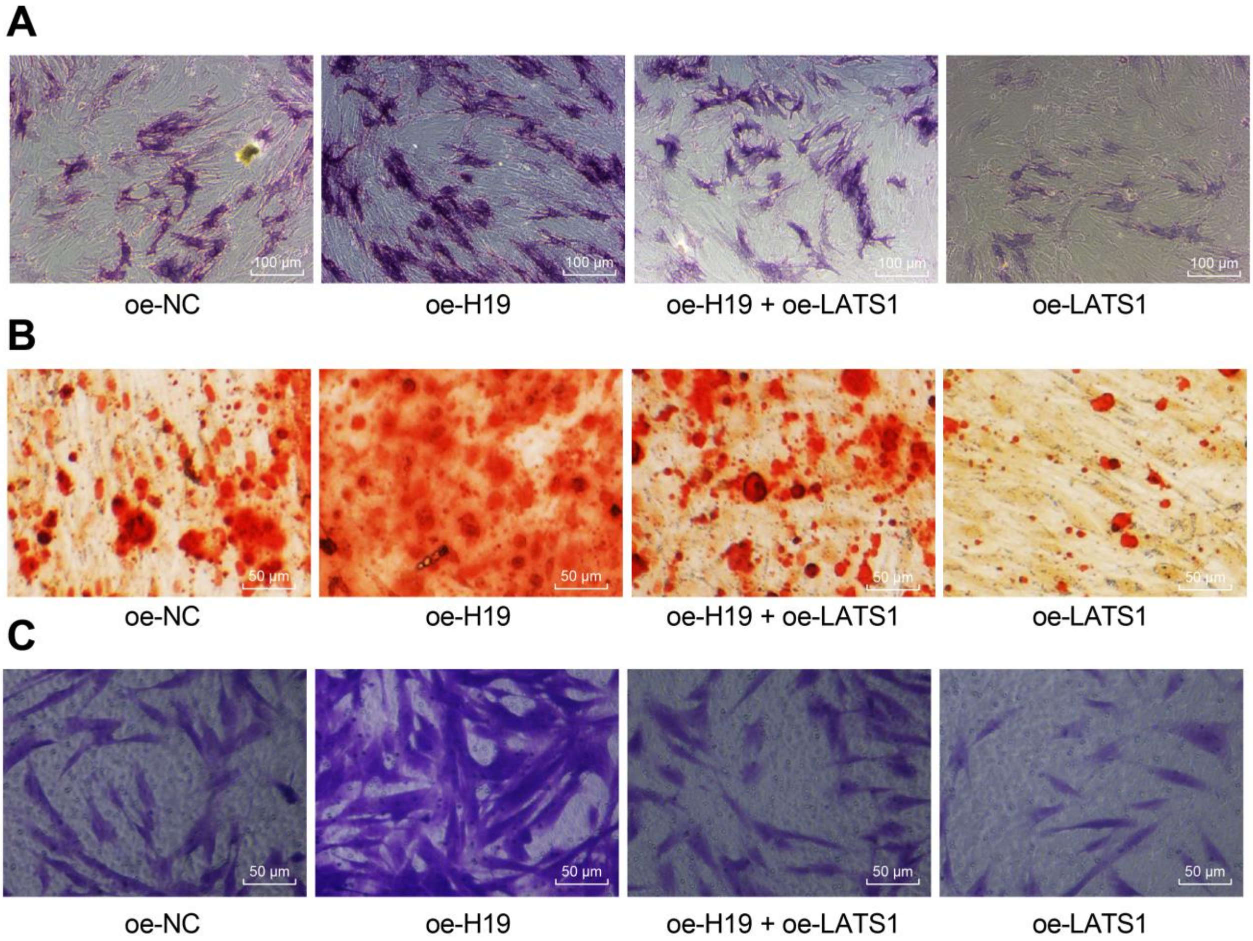

Figure S3 A, ALP staining detection (×100). B, the formation of mineralized nodules examined by Alizarin red staining (×200). C, migration of hDPSCs evaluated by Transwell assay (×200).

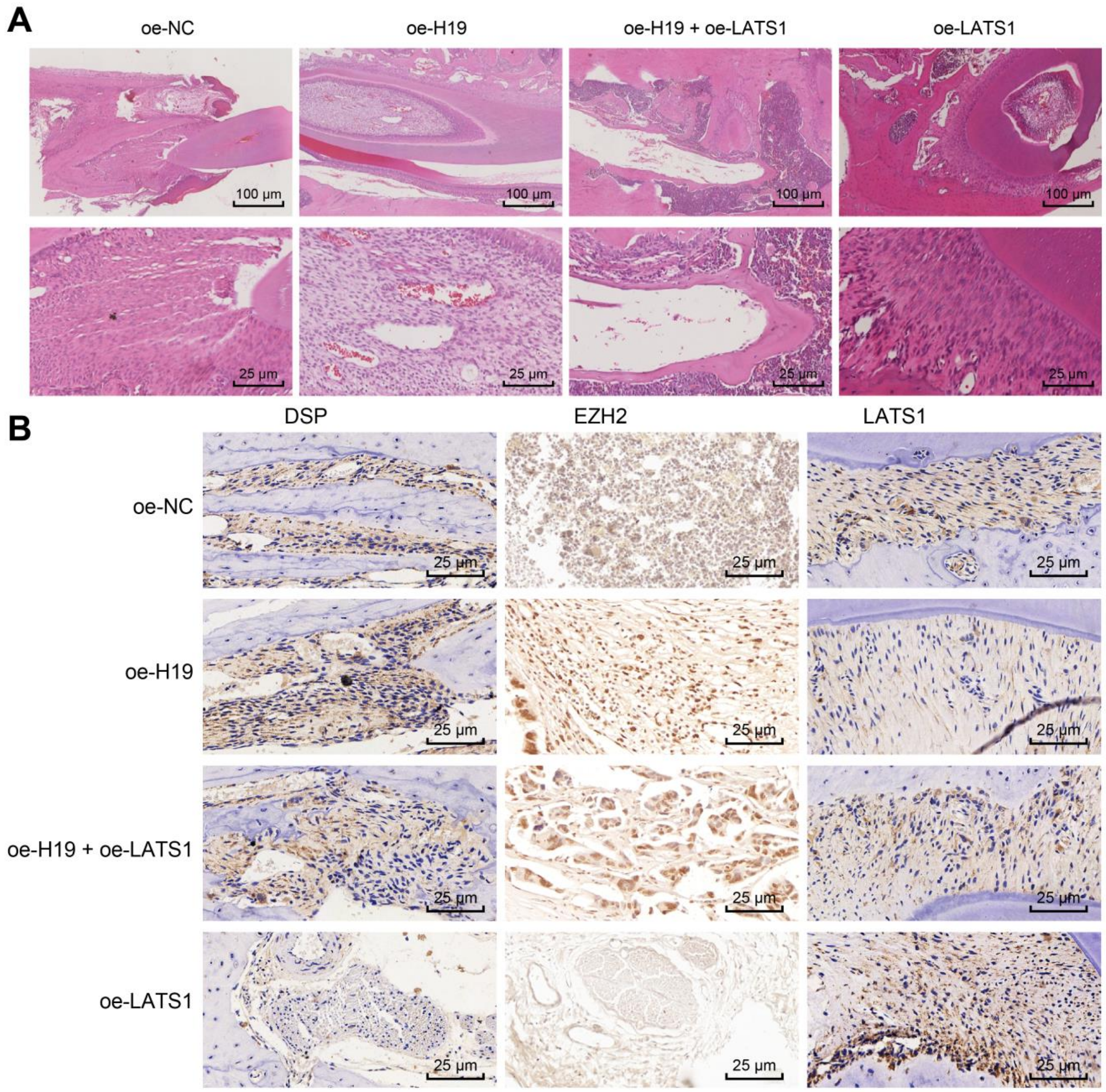

Figure S4 A, the formation of dentin and dental pulp-like cells assessed by HE staining (×100). B, the expressions of DSPP, EZH2 and LATS1 in dentin and dental pulp-like tissues evaluated by immunohistochemistry (×200).

Table S1 Primer sequences for RT-qPCR

Gene	Primer sequence
H19	F: 5'-TTCAAAGCCTCCACGACTCT-3'<br>R: 5'-GCTCACACTCACGCACACTC-3'
ALP	F: 5'-CCACGTCTTCACATTTGGTG-3'<br>R: 5'-AGACTGCGCCTGGTAGTTGT-3'
DMP1	F: 5'-ACA GCAGCTCAGCAGAGA GT-3'<br>R: 5'-TAATAGCCGTCTTGGCAGTC-3'
OCN	F: 5'-GGCAGCGAGGTAGTGAAGAG-3'<br>R: 5'-CTGGAGAGGAGCAGAACTGG-3'
DSPP	F: 5'-GTCGCTGTTGTCCAAGAAGA-3'<br>R: 5'-ATCCTCATCTGCTCCATTCC-3'
LATS1	F: 5'-CCACCCTACCCAAAACATCTG-3'<br>R: 5'-CGCTGCTGATGAGATTTGAGTAC-3'
GAPDH	F: 5'-ATGGAGAAGGCTGGGGCTC-3'<br>R: 5'-AAGTTGTCATGGATGACCTTG-3'

Notes: RT-qPCR, reverse transcription quantitative polymerase chain reaction; F, forward; R, reverse; GAPDH, glyceraldehyde-3-phosphate dehydrogenase; ALP, alkaline phosphatase; DMP1, dentin matrix protein 1; OCN, osteocalcin; DSPP, dentin sialophosphoprotein; LATS1, large tumour suppressor 1.
